# The Evaluation of Further Complications after the Extraction of the Third Molar Germ: A Pilot Study in Paediatric Dentistry

**DOI:** 10.3390/healthcare9020121

**Published:** 2021-01-25

**Authors:** Giacomo D’Angeli, Francesca Zara, Iole Vozza, Francesco Maria D’Angeli, Gian Luca Sfasciotti

**Affiliations:** 1 Department of Oral and Maxillo-Facial Sciences, Sapienza University of Rome, 00161 Rome, Italy; giac.dangeli@gmail.com (G.D.); iole.vozza@uniroma1.it (I.V.); gianluca.sfasciotti@uniroma1.it (G.L.S.); 2 Department of Anesthesiology and Intensive Care, Sapienza University of Rome, 00161 Rome, Italy; francescomaria.dangeli@uniroma1.it

**Keywords:** lower third molar, germectomy, pediatric dentistry, complications, suturing techniques

## Abstract

Some authors suggest germectomy to prevent the impaction of mandibular third molars, which can cause anterior crowding. The aim of the study, conducted with 2 years of follow-up, was to clarify when the extraction of the germ of the third molar is optimal, together with possible post-operative complications. A new surgical approach was performed through the application of a combined suture, which can provide better wound healing. The study was performed on 25 patients with a mean age of 15.44 ± 2.06. Based on orthodontic and surgical indications, 46 germectomies were performed. Follow-ups were conducted after 1 week, 2 weeks, 4 weeks, 1 year and 2 years. All procedures were carried out by the same operator and were standardized. Data analysis was conducted using R-Software. Statistical evaluation used the chi-squared test and the Monte Carlo test. The level of significance was set as 0.05. Results showed that out of 46 germectomies, the prevalence of complications was 4.2% for two patients (8%). Both complications were observed in male patients. In the first case, the patient (at Nolla stage 7) showed delayed onset infections after four weeks; in the second case, the patient (at Nolla stage 6) showed bleeding immediately after surgery and suture. With reference to delayed onset infections, no statistically significant association was found among gender (*χ^2^* = 0.719; *p* = 0.396), germ development stage (*χ^2^* = 2.595; *p* = 0.658) or Winter’s classifications (*χ^2^* = 0.046; *p* = 0.829); similarly, no significant associations were found among bleeding, gender (*χ^2^* = 0.719; *p* = 0.396), germ development stage (*χ^2^* = 2.595; *p* = 0.658) or Winter’s classification (*χ^2^* = 0.046; *p* = 0.829). From our results, it is also possible to state that post-operative complications following germectomy of the mandibular third molar germ in adolescence occur in a significantly reduced percentage of patients, so this oral surgery treatment becomes a reliable surgical technique in adolescence.

## 1. Introduction

The term germectomy refers to the extraction of a dental element during its growth, when the crowns and the roots have not yet completed their development. This is an elective surgery, performed after an accurate diagnosis and included in specific treatment plans, especially regarding mandibular third molars, which have a high probability to be impacted in accordance with their development stages [1]. Allowing the growth of the wisdom tooth is troublesome, so knowing its development stages might prevent the recurring inclusions that occur in 24–73% of adolescents in Europe [2,3]. Development of the wisdom tooth occurs inside the bone crypt at the mandibular surface; between the ages of 6 and 8, the germ is located at the inner mesial corner between the ramus and the body of the mandible, on the lower margin of the temporal crest. Between the ages of 8 and 12, the germ goes deeper into the bone and moves further from the surface. After further development, the third molar germ moves toward the center of the mandibular body and the connection with the cortical plate starts to close. At this point, the crypt of the tooth is located on the geometrical projection of the posterior extension of the line joining the vestibular cusps of the inferior teeth. Between the ages of 12 and 16, when crown mineralization is complete, roots develop; the germ is located behind the second molar, which has now erupted, and lies beneath the occlusal plane [4,5,6,7]. In the late 80′s and early 90′s, there were many opinions about etiology (the prevention and prediction of third molar impaction). From an orthodontic point of view, the main interest was the relapse of orthodontic treatment, which can be caused by an impacted lower third molar. Therefore, some authors have suggested the use of germectomy treatment to prevent inclusion of the mandibular third molars, avoiding a possible relapse of orthodontic treatment [8]. In addition, some authors have also proved a correlation between the inclusion and the number of roots of the third molar, the ramus and the alveolar bone height [2,9]. Indeed, there is greater probability of inclusion in cases where the tooth has more than two roots, the ramus has shown a lesser development or the alveolar nerve is higher [10]. On the whole, no differences between men and women regarding impaction are present, but it was demonstrated that facial characteristic and inclination of eruption can influence impaction [11,12]. Indeed, people with brachycephaly have a lower chance of having an impacted third molar than people with dolichocephaly, so the length of the mandible and the level of crowding determine whether or not the third molar remains impacted. Literature reports that, regarding inclination of the third molar, 41% are impacted with mesio-angular inclination (25% vertically and 11% horizontally), suggesting that impaction is related to the inclination of the eruption and the angle between the second and the third molar [3,13,14,15]. For all of these reasons, it can be said that many aspects influence the possible inclusion of the wisdom tooth, making it difficult to identify any indication justifying early germ extraction. Moreover, a surgical indication for an early extraction is justified only when the germ is close to the alveolar nerve [16]. In this way, it is possible to prevent further complications, such as the roots being too close to the nerve, when development of the tooth is completed, which make the surgical approach more difficult, as it comes with a major risk of post-operative complications.

Unfortunately, few recent articles show a detailed overview of intra-operative drawbacks or possible post-operative complications in cases of early extraction of the third molar germ. In light of this, it still remains a challenge to recommend an immediate approach or offer simplified management for this procedure [17,18,19].

In light of these considerations, the aim of the study was to investigate possible post-operative complications with two years of follow-up together with a new surgical approach, which was performed through the application of a combined suture, helping provide better wound healing.

## 2. Materials and Methods

### 2.1. Study Design and Methodology

A pilot clinical trial was conducted to investigate possible post-operative complications and clarify a correct management technique for the extraction of the germ of the third molar. This study used an experimental design based on the guidelines recommended by the Consolidated Standard of Reporting Trials—CONSORT 2010 [20]. The study protocol complied with the Guidelines for Good Clinical Practice, according to the Declaration of Helsinki (1975). Regarding ethical approval, the study received the protocol number (n. 2803-2017) from the Institutional Review Board of territorial NHS facilities. All patients with surgical or orthodontic indications were undergoing extraction of the germ of the third molar. In accordance with the design of the study, a surgeon performed the oral surgery operation and a different dental practitioner followed the patient during the follow-up period.

### 2.2. Sample Size Calculation

The sample size calculation was performed through G-Power analysis. The calculation was done by setting 0.05 α, 0.8 β and 0.5 effect size. In addition, the calculation of the total sample size shows a minimum number of 44 third molar extractions.

#### Dimension of Sample and Inclusion Criteria

Twenty-five patients were referred to the Oral and Maxillo-Facial Sciences Department, Pediatric Dentistry Unit, University Hospital Policlinico Umberto I, “Sapienza” University of Rome from December 2017 to September 2018.

Since some patients needed a bilateral extraction, 46 germectomies were performed after prior radiographic evaluation. The sample consisted of 15 males (60%) and 10 females (40%), with a mean age of 15.44 ± 2.06 (range 11–17 years old). Inclusion criteria were: an age between 11–17 years old, the presence of lower third molar germs (5th, 6th, 7th and 8th stage of development according to Nolla’s classification), the absence of systemic diseases and signed consent by the legal guardian of each subject. The exclusion criteria were: the absence of lower third molar germs or their early growth (1st, 2nd, 3rd and 4th stage of development according to Nolla’s classification), the absence of surgical or orthodontic indication for the potential extraction of the third molars germ, the presence of osteolytic lesions associated with the lower third molar germ, the absence of the second molar, the presence of systemic diseases or possible contraindications or the lack of signed consent by the legal guardian of each subject. Crowding and malocclusion were not discriminating parameters during the enrollment process; indeed, any malocclusion was excluded and patients with or without crowding were included.

In accordance with the aim of the study, all germs were classified based on Winter’s classification and Nolla’s stages [21,22,23]. Winter’s classification gave information about the inclination of the third molar germ compared to the longitudinal axis of the second molar. In accordance with the article of Barroso et al., this classification was used to better understand the surgical approach (which is simpler when the germ has a mesio-angular position) [21]. Alternatively, Nolla’s classification was used to standardize the stage of development of the third molar germ (from 1st to 8th). In this way, it was possible to understand how best to time the surgical operation.

In addition, the following parameters were considered to evaluate possible post-operative complications: swelling, delayed onset infections, bleeding, alveolar osteitis, paresthesia of the inferior alveolar nerve (IAN), paraesthesia of the lingual nerve (LN), second molar restoration damage, pain and severe trisma (Table 1 and Table 2) [24,25].

### 2.3. Surgical Protocol

All procedures were carried out by the same operators and with the same assistants, and all instruments and surgical protocols were standardized.

**Figure 1:**
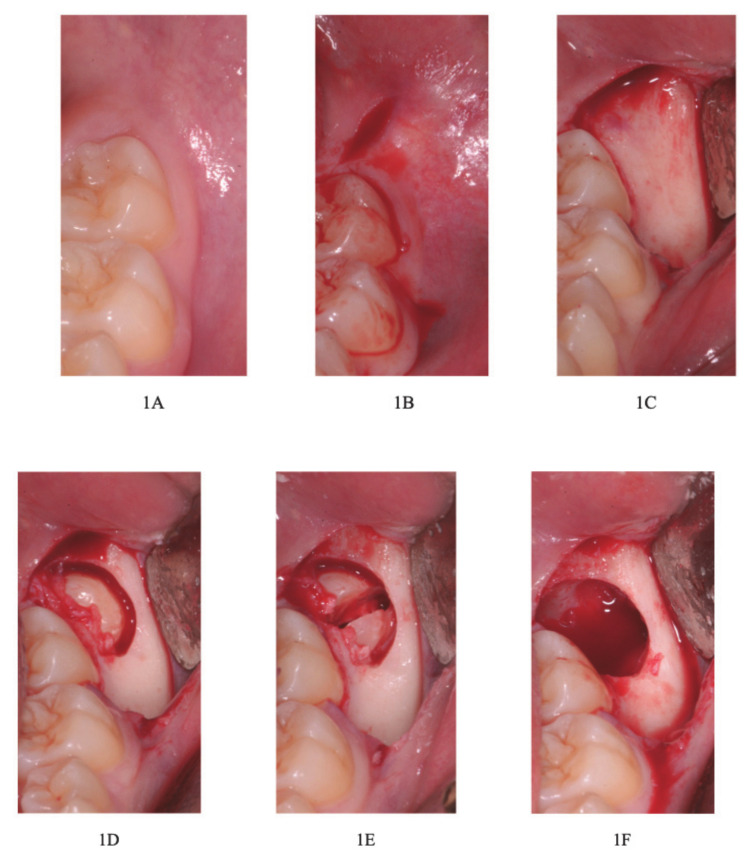

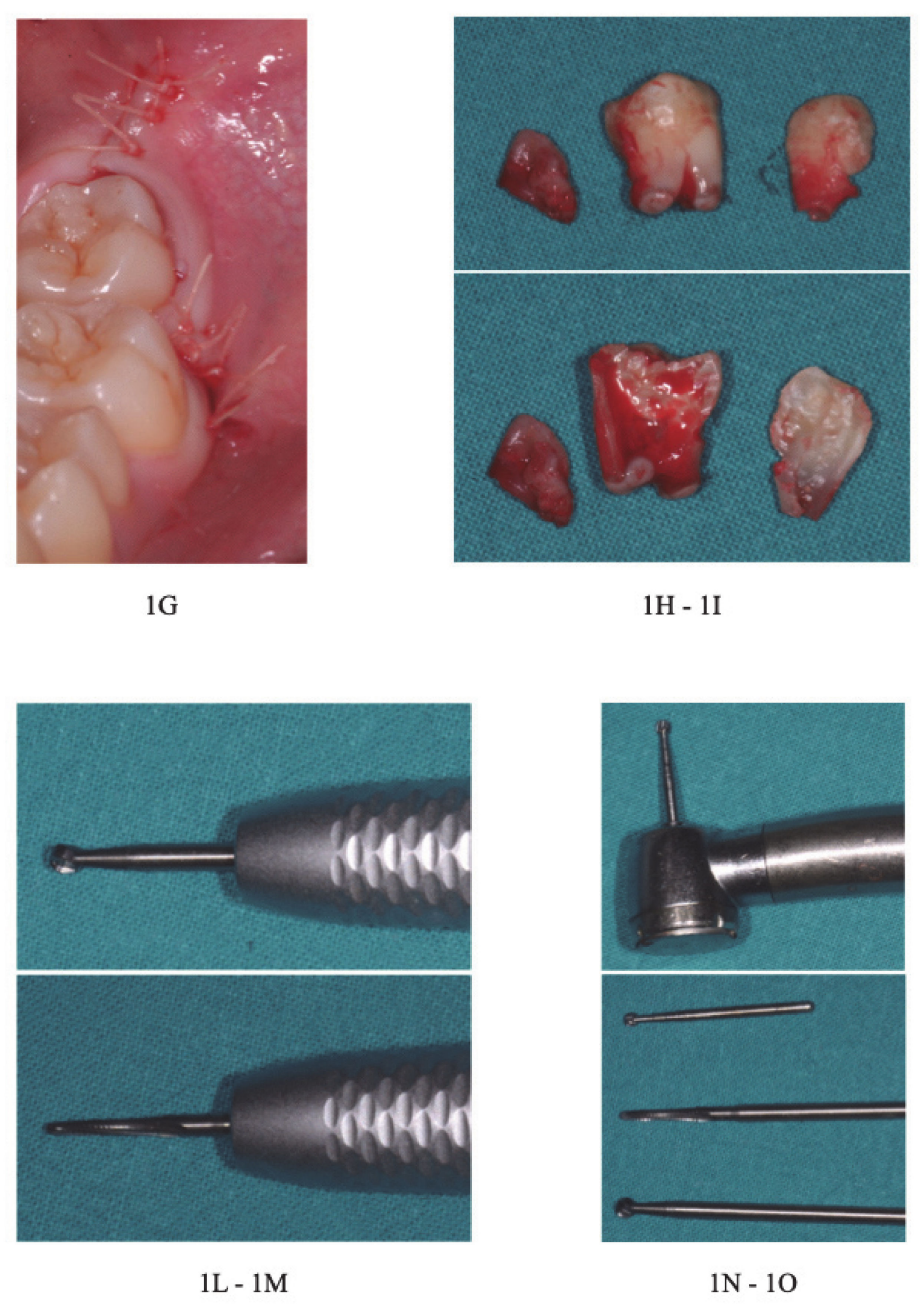
(**A**) Clinical examination; (**B**) flap incision; (**C**) flap elevation; (**D**) ostectomy with ball and fissure burs; (**E**) odontectomy with spherical bur; (**F**) avulsion; (**G**) suture (absorbable suture Ethicon Vicryl, Rapide in polyglactin 910, with a needle 3/8 of 19 mm, USP 3/0—white).; (**H**–**I**) avulsion of the fragments of the germ; (**L**): straight handpiece with the ball bur; (**M**) straight handpiece with the fissure bur in carbon tungsten; (**N**) high velocity handpiece with spherical bur; (**O**) the ball, fissure and spherical burs.

Firstly, mandibular nerve-block anesthesia was conducted though the use of 0.9 mL of local anesthesia without using vasoconstrictors and 0.9 mL of local anesthesia with a vasoconstrictor located at the buccal nerve. Secondly, a mucoperiosteal flap was incised and elevated, followed by ostectomy of the vestibular cortex with a fissure bur in carbon tungsten. The germ of the mandibular tooth was always dissected and its odontectomy was performed with a spherical bur on a high velocity handpiece. We proceeded with an avulsion of the germ fragments through college pliers or hemostat pliers (Klemmer or kelly curve) or similar tools. The surgery ended with revision and a saline solution rinse of the cavity followed by application of the suture (absorbable suture Ethicon Vicryl, Rapide in polyglactin 910, with a needle 3/8 of 19 mm, USP 3/0—white) (Figure 1A–O).

#### Description of the Combined Suture

As per the aim of this article, a combined suture was tested to evaluate the wound healing process. Indeed, if the second molar was partially erupted or possessed disto-angular inclination, it would be important to stabilize the flap beneath the second molar’s equator to counter muscular forces. An oblique suture point was created in order to connect the vestibular and lingual papillae distal to the second molar.

**Figure 2:**
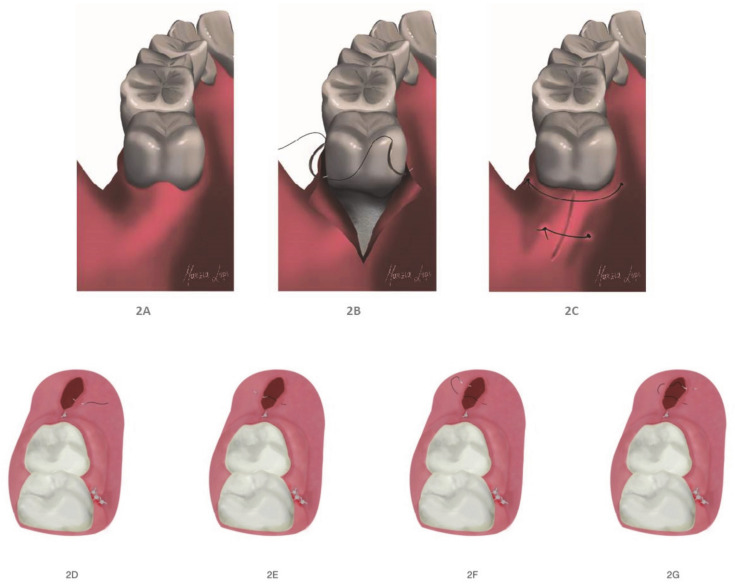
(**A**,**B**) The pre- and post-modified interrupted suture; (**C**) the modified interrupted suture; (**D**–**G**) the external horizontal mattress suture.

The needle was inserted from the vestibular side (2mm from gingival margin) to oral side (slightly more than 2 mm) of distal papilla. The distance from the gingival margin was 2mm and stitched from lingual direction to vestibular direction to stabilize the flap (Figure 2A–C).

In addition, a Donati suture was performed by inserting the needle from the outer or mucosa side of the first flap, 2–4 mm from the line of incision. The needle was then turned on a plane parallel to the incision margin and with a “U” motion (Figure 2F), re-entering from the lingual mucous side. In this way, it penetrates more apically from the lingual side, inverting the vestibular flap. This kind of suture crosses the space between the two flaps, emerging on the mucous side of the first flap at the same distance from the free margin adopted for the first perforation [26] (Figure 2D–G).

According to the post-operative protocol followed after the operation, antibiotics (1 g amoxicillin + clavulanic or macrolide antibiotic when the patient had an allergy to a penicillin) were administered over the following six days, pain killers were given in accordance to patients’ necessity, and Chlorhexidine 0.2% (Corsodyl spray) was given from 24 h to 7 days after the operation, 2 times a day.

Patients were informed not to rinse the mouth or spit for the first 24–48 h to stabilize the clot [27,28]. In addition, a liquid diet for the first 3 days was prescribed, together with specific oral habit indications.

### 2.4. Statistical Methodology

Data analysis was conducted using R-Software. The associations between the two complications observed, the gender of patients, the germ development stage (Nolla) and Winter’s classification were evaluated using a chi-squared test and a Monte Carlo test. The level of significance was set as 0.05.

## 3. Results

All patients were put in a follow-up program intended for suture removal after 1 week and meant to check wound healing and possible further complications after 2 weeks, 1 month, 1 year and 2 years [29,30]. Of the 46 extracted third molar germs, the prevalence of complications was 4.3% (2 complications out of 46 germectomies, identified in the Table 3 with “V”), occurring in 8% of patients. Two complications were observed in two different male patients (Table 1), according to Winter’s classification (Table 2). In the first case, the patient, whose germ had been classified as Nolla stage 7 (Table 3), showed delayed onset infections after one month (Table 4).

At the site of infection, the abscess was drained of purulent material (and treated with antibiotics for a week). In the second case, the patient, whose germ has been classified as Nolla stage 6 (Table 3), showed bleeding immediately after surgery and suture (Table 4).

The associations between the two complications (delayed onset infection and bleeding) and patient gender, germ development stage (Nolla’s classification) and Winter’s classification were investigated (Table 5 and Table 6).

During surgery, the second molar crown was never damaged. At the one-week follow-up visit to remove the sutures, patients did not show swelling or pain. At the following visits, no cases of alveolar osteitis, lingual nerve and inferior alveolar nerve paresthesia or trismus were reported.

## 4. Discussion

The results of this study confirm a low presence of complications when the oral surgery treatment is performed when the germ is in the 5th, 6th or 7th of Nolla’s stages. The study also proves the efficacy of the modified suture that was tested in the sample, as it provided good wound healing.

The almost non-existent percentage of complications is certainly linked to the value of Nolla’s classification, a correct surgical technique, the skills of the operator and the compliance of the patient in accurately following the post-operative instructions [18,19,29]. Age might be the most significant factor influencing pre- and post-operative management. Indeed, several studies have shown that adolescents have less surgical and post-operative complications, together with a faster recovery [14,17,19].

Literature confirms and supports the concept of post-operative risks, which can increase when there is a delay in surgery, leading to development of a germ with an higher bone density and a complete root development. Therefore, when patients get older, third molar extraction becomes more difficult and the procedure needs more time. Data show that the risk of complication after third molar extraction is 1.5 higher for patients over the age of 25 [31].

Chiapasco et al. [19] have analyzed and compared complications and side effects after 1500 impacted third molar extractions among three groups of patients by age [19]. Group A was classified as 9–16 years old, group B as 17–24 years old and group C as over 24 years old. This study did not show any significant difference between group A and B, while complication rate and side effects rate increased in group C. In particular, group C experienced neurological lesions, which can be very debilitating, have further therapeutic consequences and incur further necessities of care. The increase in complications and side effects was correlated to patients’ age, as shown by the results of group C. These results are in accordance with what was explained in our study and our statement that germectomy should be performed after careful cost–benefit analysis and when specific indications exist [32,33,34].

Ganss C. et al. [35], in their retrospective study, evaluated the delayed-onset infection rate correlated to the distal space of the second molar. The ratio between the distal space and the crown width, both measured according to Ganss’s protocol on panoramic radiographies, was obtained for 218 germectomies performed due to orthodontic reasons in 134 patients. They observed 20 cases of infection out of 218 germectomies 2–8 weeks after the surgery. The fact that most infections occurred after 4 weeks post-surgery, when the effects of antibiotics and chlorhexidine oral rinse terminate (as our experience also reported), shows that the most important risk factor is the anatomical factor. The delayed infection rate in this study is higher than the immediate infection rate [36,37,38,39,40,41,42,43]. This low infection rate might be related to surgical technique, operator experience or treatment with antibiotics—all factors which have small to negligible effects at 4 weeks after the operation, when the infection started.

Another study has shown that, among 1151 patients with symptoms, pain was the most common symptom, followed by swelling, oral discomfort and purulent drainage. [44] The incidence rate of said complications increased with age [45].

A prospective study evaluated surgical and post-operative complications in 9574 patients, for whom 16,127 third molars had been removed. The removal of the mandibular third molar during adolescence induced a reduced operative and post-operative morbidity. The study showed that an increased number of complications (alveolar osteitis, infection and dysesthesia) occur as a result of the removal of third molars from older patients. This study suggests that, when indications to do so exist, third molars should be removed during adolescence, thereby reducing the incidence of post-operative morbidity [46].

Regarding injuries to the IAN and LN, Rui Sun et al. investigated the characteristics of the adjacent anatomy of the mandibular third molar germs (MTMG) in their study [47,48]. Patients enrolled in the study, aged between 12 and 17 years, underwent cone–beam computed tomography (CBCT) and, subsequently, the authors analyzed structures and parameters by comparing them with age, sex, degree of tooth development and tooth position [49]. The chances of alteration of the cortical profile of the inferior alveolar canal (IAC) or of the hard tissues of the germ in contact with the IAC were significantly lower in the 12 to 13 year old group and when the Nolla stage ≤ 6, i.e., when the germ had just completed mineralization of the crown and root development began. Anatomical features studied by CBCT suggest that the risk of IAN and lingual nerve injury were lowest in the 12 to 13 year age group during MTMG removal. Anatomical factors for IAN injury were: the location of the MTMG relative to the IAC, degree of inclusion and root development. [50]

Among the relationships between MTMG and IAC, IAC cortical integrity was an important factor in predicting postoperative IAN paraesthesia, and the degree of cortical interruption was positively correlated with IAN injury [51].

Regarding lingual nerve injury, the CBCT can only show the thickness of the lingual bony cortex, and the lingual nerve cannot be shown in the CBCT image. Therefore, the risk of lingual nerve injury is often assessed through observation of the lingual cortex. The main anatomical factor evaluated for lingual nerve damage is the perforation of the lingual cortex because the loss of lingual bone cortex in the retromolar area would have provided for vulnerability of the lingual nerve during surgery. Perforation was often positively associated with the angulation of mature rooted teeth [52]. After CBCT analysis, the authors concluded that, in order to avoid a higher chance of damage to the IAN, germectomies should be performed in the age range of 12 to 13 years old. In their retrospective study, Zhang Z.-Q. and Zhang Q.-B. evaluated the effects of early extraction of the immature lower third molar on the prevention of complications, particularly nerve injury following removal of the lower third molar [53]. Patients were grouped based on age and radiographic findings: group A ((mean age 17 years) immature teeth with non-closed apical foramen); group B, (mean age 39 years). mature teeth with closed apical foramen). In group A, the incidence of complications was very low (all complications were short-term), with no nerve damage. In group B, the incidence of complications was greater, with the presence of nerve lesions. All complications were temporary, except for two permanent (>6 months) inferior alveolar nerve numbness complications. In this study, early removal of the lower third molar was effective in avoiding some post-operative complications, particularly nerve injuries. Early extraction of the lower third molar in young people is recommended after a team consultation.

A study of 4004 patients showed a 1.5 times greater probability of complication if the patient had their impacted third molar extracted over the age of 25, with general risk increasing with age up to 65 years [54,55]. Similarly, in a study of 583 patients, age was related to risk of complication [56]. Other studies also show that post-operative risks rise in frequency with increasing age [16,57,58,59,60]. In a study in which germectomy was performed on 300 teeth in patients aged 12–19 years, no lingual nerve injury occurred [61]. The 2007 white paper from the American Association of Maxillofacial Surgeons concluded that early third molar removal may be associated with a lower incidence of morbidity and less financial hardship for the patient [45,62].

A 2016 Cochrane review examined the prophylactic extraction of asymptomatic impacted third molars with respect to preservation in adolescents and adults. Searching the electronic database resulted in insufficient evidence to support or refute the need for routine prophylactic removal of asymptomatic wisdom teeth. Cochrane concluded that patient values and clinical skills should guide shared decision making with patients who have asymptomatic impacted wisdom teeth [63].

## 5. Conclusions

Based on the results obtained from this clinical study, it is possible to state that there are no significant associations between gender or germ development according to Nolla or Winter’s classification and post-operative complications. Moreover, the combined suture, which was tested in this study, proved to be effective, helping the wound healing process and avoiding gingival hypertrophy to the distal surface of the second molar.

Therefore, germectomy is becoming a reliable surgical technique with advantages that must be used when there are surgical and orthodontics indications to do so. Despite the results obtained, further studies, with a bigger sample, are necessary in order to better evaluate possible post-operative complications and compare this combined suture with other kinds.

## Figures and Tables

**Table 1:** Complications according to patients’ sex.

COMPLICATIONS	MALES *N* = 27 teeth (%)	FEMALES *N* = 19 teeth (%)
Swelling	0%	0%
Delayed oneset infections	1 (3.7%)	0%
Alveolar Osteitis	0%	0%
Paresthesia of IAN	0%	0%
Lingual Paresthesia	0%	0%
Bleeding and Hemorrhage	1 (3.7%)	0%
Pain	0%	0%
2 molar restoration damage	0%	0%
Severe trisma	0%	0%

**Table 2:** Complications according to the position of the impacted mandibular third molars (Winter’s classification). B: mesio-angular position; C: vertical position [21].

COMPLICATIONS	B *N* = 44 teeth (%)	C *N* = 2 teeth (%)
Swelling	0%	0%
Delayed oneset infections	1 (2.3%)	0%
Alveolar Osteitis	0%	0%
Paresthesia of IAN	0%	0%
Lingual Paresthesia	0%	0%
Bleeding and Hemorrhage	1 (2.3%)	0%
Pain	0%	0%
2 molar restoration damage	0%	0%
Severe trisma	0%	0%

**Table 3:** Complications observed during the various checks following the surgery.

COMPLICATIONS	Immediately after the Germectomy	1 week	1 month	1 year	2 year
Swelling	X	X	X	X	X
Delayed oneset infections	X	X	V	X	X
Alveolar Osteitis	X	X	X	X	X
Paresthesia of IAN	X	X	X	X	X
Lingual Paresthesia	X	X	X	X	X
Bleeding and Hemorrhage	V	X	X	X	X
Pain	X	X	X	X	X
2 molar restoration damage	X	X	X	X	X
Severe trisma	X	X	X	X	X

**Table 4:** Complications according to Nolla’s classification of the impacted mandibular third molars. 11 third molars were in stage 5, 13 were in stage 6–7 and 9 were in stage 8.

COMPLICATIONS	5	6	7	8
Swelling	0%	0%	0%	0%
Delayed oneset infections	0%	0%	1 (7.7%)	0%
Alveolar Osteitis	0%	0%	0%	0%
Paresthesia of IAN	0%	0%	0%	0%
Lingual Paresthesia	0%	0%	0%	0%
Bleeding and Hemorrhage	0%	1 (7.7%)	0%	0%
Pain	0%	0%	0%	0%
2 molar restoration damage	0%	0%	0%	0%
Severe trisma	0%	0%	0%	0%

**Table 5:** Association between delayed onset infection and gender, Nolla’s classification and Winter’s classification.

Delayed Onset	*χ^2^*	*Df*	*P*
Gender	0.719	1	0.369
Nolla’s classification	2.595	3	0.658
Winter’s classification	0.046	2	0.829

**Table 6:** Association between bleeding and gender, Nolla’s classification and Winter’s classification**.**

Bleeding	*χ^2^*	*Df*	*P*
Gender	0.719	1	0.369
Noll’s classification	2.595	3	0.658
Winter’s classification	0.046	2	0.829

## Data Availability

Data and methods used in the research were presented in sufficient detail in the paper so that other researchers can replicate the work. Raw data must be publicly available.
